# Increased Perceptual and Motor Performance of the Arms of Elite Water Polo Players

**DOI:** 10.1155/2019/6763470

**Published:** 2019-02-05

**Authors:** Jovan Gardasevic, Selcuk Akpinar, Stevo Popovic, Dusko Bjelica

**Affiliations:** ^1^Faculty for Sport and Physical Education, University of Montenegro, Niksic 81400, Montenegro; ^2^Physical Education and Sport Department, Faculty of Education, Nevşehir Haci Bektas Veli University, Nevşehir 50300, Turkey

## Abstract

**Background:**

It has been stated that long-term participation in sport training can influence the motor asymmetry of the arms with a decreased interlimb difference. However, whether this pattern is observable in different sports and with different variables, like perceptual performance, still needs to be tested. Therefore, we investigated if long-term sports participation might modify the motor and perceptual performance asymmetries of arms in water polo players. It was hypothesized that water polo players would perform with less interlimb asymmetry in comparison to nonathletes.

**Methods:**

Right-handed water polo players and nonathletes were tested on motor performance for both arms during a reaching task. Thirteen water polo players and thirteen nonathletes performed reaching movements under two experimental conditions: (a) right arm and (b) left arm. Velocity, accuracy, hand path deviation from linearity, and reaction time were calculated for each trial and for both arms. The potential interlimb differences in movement performance could be assessed by testing.

**Results:**

Consistent with the hypothesis, our findings showed that water polo players displayed substantially less asymmetry in the performance of accuracy and reaction time.

**Conclusions:**

These findings suggest that performance asymmetries of arms can be altered via intense long-term practice.

## 1. Introduction

Approximately 90% of people are right-handed [1, 2]. Many everyday tasks involving tool use, such as writing with a pencil and using a key, tend to show a right-hand preference. Arm preferences were measured to reach an object extensively located in the working place under different sensory and motor situations [3]. Supporting this notion, coordination and accuracy of movements of the left and right arms were found to be distinctively affected by vision and no-vision situations [3–5]. Specifically, right arm accuracy advantage diminishes when the visual feedback is occluded. Moreover, the coordination pattern of the right arm performance is abundantly reduced with visual occlusion. In accordance with these changes, the selection of the right arm in the contralateral space was considerably reduced, which was resulted with the more left arm preference in its own side when participants were asked to reach targets under no-visual feedback condition [6]. These results suggested that arm preferences reflect decisions that are based on performance differences between the arms, which change with different task conditions. This experience-dependent plasticity in limb selection, supported by an array of previous studies [6, 7], suggests that long-term training, in particular in motor tasks, might lead to systematic changes in performance asymmetries. For instance, Teixeira and Okazaki [8] and LA Teixeira and MCT Teixeira [9] contributed some evidence that hand selection was modified by unimanual exercise with the left hand.

A study by Coelho et al. [7] suggested that a more frequent usage of the right arm compared to the left arm in the working space may be related to having better coordination and accuracy of the right arm. It has been also suggested that the selection of which hand to be used can be affected by intensive training. Mikheev et al. [10] found more left arm preference in right-handed elite judoists than nonathletes. As the judoists use more bimanual hand movements for attack and defence, it may be reasonable for them to prefer their left arm more compared to nonathletes. In line with this proposition, some researchers reported that motor preferences, as well as the cortical representations of the body, are not predetermined entities and can be adapted through experience such as sport or musical practice [11]. A recent study by Maeda et al. [12] also showed a modification of hand preference between kung fu experts and amateurs, displaying a weaker strength of right-hand selection across kung fu experts compared to the hand selection of amateurs. The authors also found a less asymmetry between arms for some movements specific to kung fu for experts but not for amateurs. Moreover, it has been recently tested whether participation in unimanual [13] and bimanual sports [14] would modify performance asymmetries and, therefore, hand preference. The results for the unimanual sport (fencing) displayed considerably less interlimb asymmetry in the performance of the arm reaching for fencers in comparison to nonfencers. Similar results were found in a bimanual sport (rowing), in which a significantly lower asymmetry among arms in reaching performance in rowers was also observed in comparison to nonathletes. Furthermore, Akpinar [15] tested if extensive basketball training modifies an interlimb difference. Basketball players displayed better performance in comparison to nonathletes in accuracy and hand path deviation from linearity. In addition, Youngen [16] found that female athletes are significantly faster than female nonathletes in movement speed and reaction time. Thus, one of the main variables that can be a good predictor of being a successful athlete is the reaction time [17].

Many sports require a high level of perceptual and motor skill acquisitions. Those requirements are even more important when the person execute the skills under different situations, like in the water. Thus, in this study, we intended to examine if long-term water polo training alters the interlimb difference for a task requiring perceptual and motor performances. To assure that our water polo players experienced long-term and intense training, we recruited members of the national team of Montenegro, U-21, where water polo is a traditional and successful sport. We predict that such long-term and intensive water polo practice, which focuses on both arms' motor performance, required to reach and maintain this elite level should result in more efficient trajectories with greater accuracy and reaction time for both arms, as compared to age-matched nonathletes. According to the results of Akpinar et al. [15, 13] and because water polo includes mainly unimanual (shooting) and some bimanual (swimming, blocking) movements, it has been hypothesized that extensive practice should increase the motor and perceptual performance of both arms and decrease the interlimb difference among water polo players. Please note that previous studies mainly focused on the effect of extensive practice on mainly motor performance asymmetries [12–15] and the novelty of the current study is the inclusion of a perceptual requirement during a motor task, which was not tested earlier.

## 2. Materials and Methods

### 2.1. Participants

Thirteen healthy male water polo players aged 18-20 years (mean = 18.7; SD = 0.75) and thirteen healthy male nonathletes aged 18-20 years (mean = 19.4; SD = 0.66) voluntarily participated in this study. All participants signed the consent form approved by the Institutional Review Board of the University of Montenegro, which was in accordance with the Declaration of Helsinki as amended by the World Medical Association Declaration of Helsinki [18]. The water polo players' experience ranged from 7 to 10 years (mean = 8.5; SD = 2.04), and they are all currently playing in the U-21 Montenegrin National water polo team. Nonathletes reported no participation in any sports. All participants of both groups reported right-handedness and scored above 65% on the extended 35-item handedness questionnaire [19], which is similar to the widely known Edinburgh Inventory [20].

### 2.2. Experimental Setup

**Figure 1:**
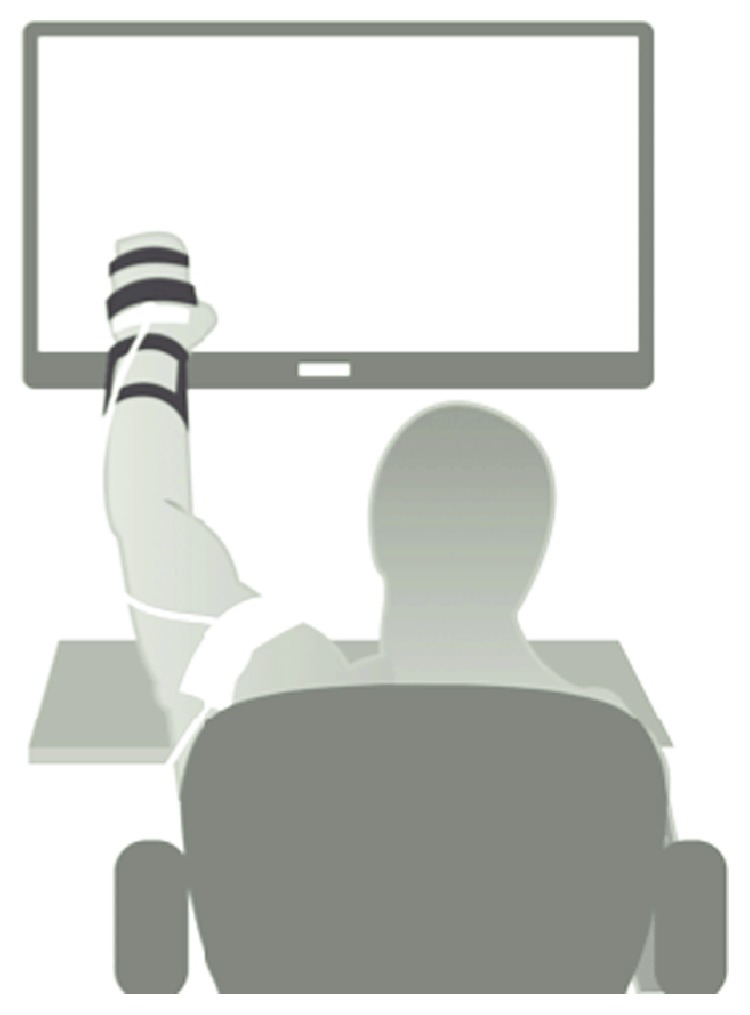
A participant seated at an adjustable chair with a sensor of the electromagnetic movement tracker attached to his left forearm.

The participants were seated at an adjustable chair with the sensor of the electromagnetic movement tracker (TrackSTAR, Ascension Technology, USA) attached to their right or left forearm, depending on which arm will be measured (Figure 1). This setup is assured reaching in the 2D horizontal space in front of the participant. One cursor, one start position for each hand, and targets were projected from a 55^″^ flat screen TV, which displayed a custom virtual reality interface. The cursor represented the index finger of the arm, and its position on the TV was updated in real time that was 100 Hz. The TV was vertically placed on a table approximately 2 m away from the participant and 1 m high from the ground. Finger displacement data were recorded at 100 Hz during the participants' movements.

### 2.3. Experimental Design

**Figure 2:**
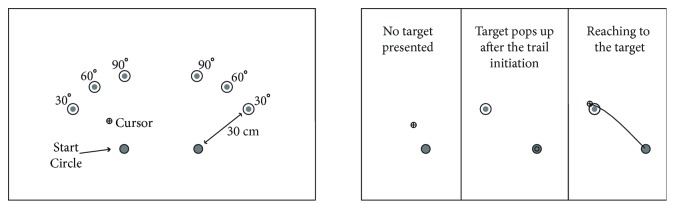
Experimental design.

Three different targets in different directions (30°, 60°, and 90°; see Figure 2) were presented (one of them for each trial) to the participants to reach from a start position. The start position was displayed as a 2 cm diameter circle and placed 20 cm away from the body midline (sternum) to the left or right side for each arm. Each target was displayed as a 3.5 cm diameter circle. The cursor was displayed as a 1.6 cm diameter circle with crosshair representing the tip of an index finger. The distance between the start position and target was set to 30 cm so that each participant could reach the target easily. The targets were not displayed before the initiation of the trial. After positioning the cursor in the start circle for 300 ms, the audiovisual “go” signal was provided, and then a target (one of the three targets) appeared on the screen, and these triggered participants to move to the target. Each target was displayed after the participant put the cursor in the start circle, so it was a restricted-pace trial. Thus, the target cannot be seen earlier, and the participants could see and plan the movement after the “go” signal. Participants could take a break during the experiment to avoid fatigue. In order to avoid the interlimb transfer, two sessions were applied. Participants performed the task either right or left arm in one session, and they came to lab to test the other arm in the second session (at least a three-day gap between sessions).

### 2.4. Experimental Task

Participants were asked to perform 60 reaching movements in a vertical plane (20 per target) from the start circle (2 cm in diameter) that represented the starting position to the target (3.5 cm in diameter), which were presented in a randomized order. Participants were instructed to reach for the target rapidly while maintaining accuracy and to stop on the target with no additional corrections. Each trial was 1 sec and was began with an auditory signal after the cursor (1.25 cm in diameter crosshair) was held in the start circle for the duration of 0.3 sec. Accuracy was rewarded with 10, 3, and 1 points for landing within 3.5, 4.5, and 5.5 cm diameters from the midpoint of the target, respectively.

### 2.5. Data and Statistical Analysis

To determine interlimb differences in the quality of movement performance, we quantified four dependent measures: (1) movement velocity, (2) movement accuracy (final position error (FPE)), (3) movement quality (hand path deviation from linearity (HPDL)), and (4) reaction time (RT). The final position error (FPE) was defined as the Euclidian distance between the center of the target and the 2D final position of the tip of the index finger. The hand path deviation from linearity (HPDL) was characterized as the ratio between the minor and the major axes of the hand path. The major axis was assigned as the longest distance between any of two points on the hand path, and the minor axis was assigned as the shortest distance perpendicular to the major axis. The reaction time (RT) was defined as the elapsed time between the presentation of a target on the TV screen and the initiation of the movement to that target. The collected data were analysed using Matlab software, and the accuracy and linearity of each reaching movement were calculated.

A three-way mixed model ANOVA (target directions; 30°, 60°, and 90° × group; water polo players and nonathletes × arms; right and left) was used to investigate whether water polo players have a less interlimb difference at one of three different targets compared to nonathletes. Before running the statistical analysis, the assumptions for MANOVA have been checked and found out that we did not meet the criteria for multicollinearity. Thus, the three-way mixed model ANOVA was conducted for each dependent variable; thus, four different statistical analyses were applied. Post hoc analysis was conducted using a Bonferroni adjustment. Assumptions for the mixed model ANOVA were checked before running the analysis for each dependent variable, and no violations were found. The significance level was tested at *p* < .05.

## 3. Results

Both groups, water polo players and nonathletes, made reaches to the three different targets located across the vertical space in front of their bodies with their dominant and nondominant arms. We have a total of four different variables to compare between water polo players and nonathletes and between the arms. Each dependent variable was displayed differently with the subtitles in this section.

### 3.1. Movement Velocity

**Figure 3:**
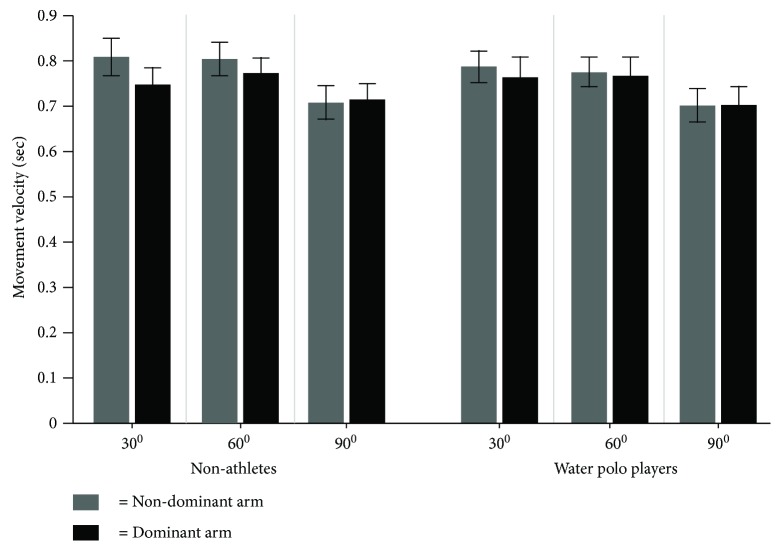
The average value of movement velocity for each target for the dominant and the nondominant arms across water polo players and nonathletes.

Figure 3 shows the average magnitude of the movement velocity for each target for the dominant and the nondominant arms across water polo players and nonathletes. As can be seen in Figure 3, the movement velocity looks very similar for both water polo players (0.75 m/s) and the control group (0.75 m/s).

**Figure 4:**
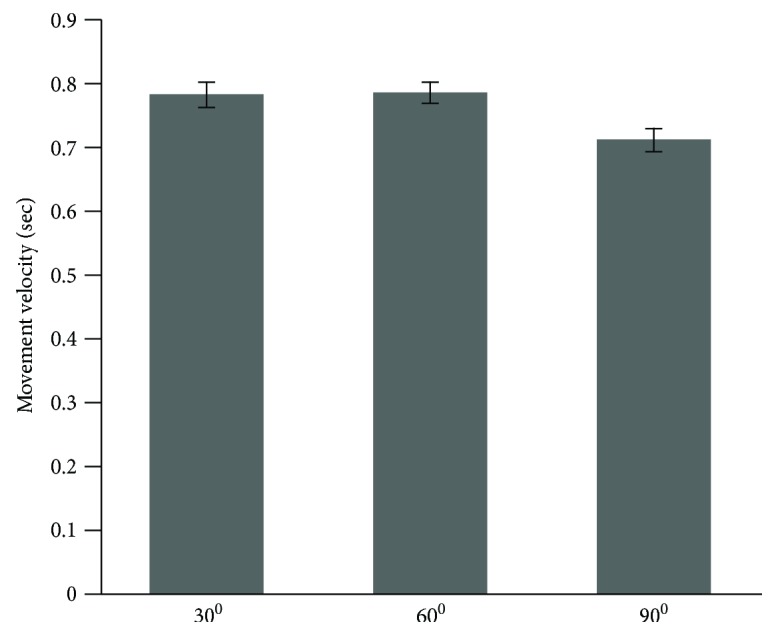
The average value of movement velocity among three targets.

The result of the statistical analysis for the movement velocity displayed only a significant difference for the target main effect (*F*(1, 24) = 91.86, *p* = .0001, *η*^2^ = .81). The post hoc analysis for the target main effect showed that targets located on 30 degrees (*M* = 0.77 and SD = 0.14 m/s) and 60 degrees (*M* = 0.78 and SD = 0.12 m/s) were reached faster than the target located on 90 degrees (*M* = 0.71 and SD = 0.13 m/s) (please see Figure 4). Normally, movement velocity can affect the movement accuracy and linearity. In our case, there was no group difference on movement velocity; thus, velocity did not affect the selected dependent variables.

### 3.2. Final Position Error

**Figure 5:**
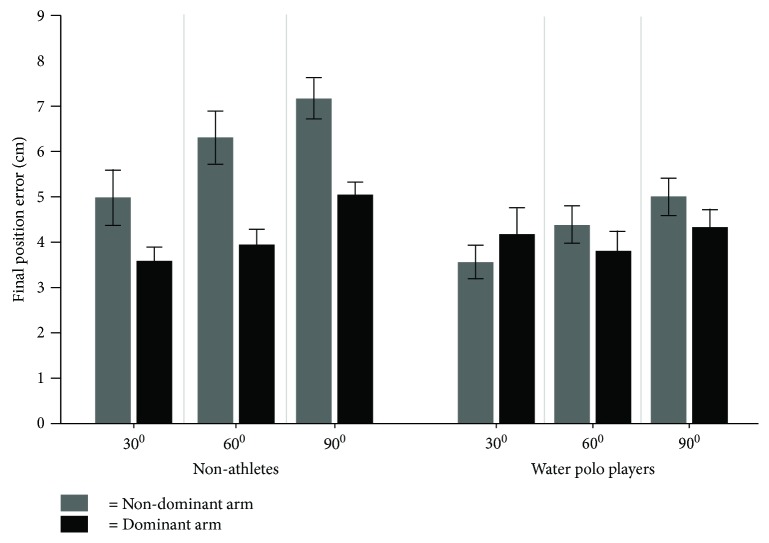
The average magnitude of the final position error (FPE) for each target for the dominant and the nondominant arms for water polo players and nonathletes.

Figure 5 shows the average magnitude of the final position error (FPE) for each target for the dominant and the nondominant arms across water polo players and nonathletes. As can be seen from the figure, water polo players almost showed similar accuracy performance for both arms for three targets. However, this pattern was not observed for nonathletes. Nonathletes showed better accuracy performance for the dominant compared to nondominant arm for all targets. Moreover, water polo players' accuracy performance seems better than that of nonathletes.

**Figure 6:**
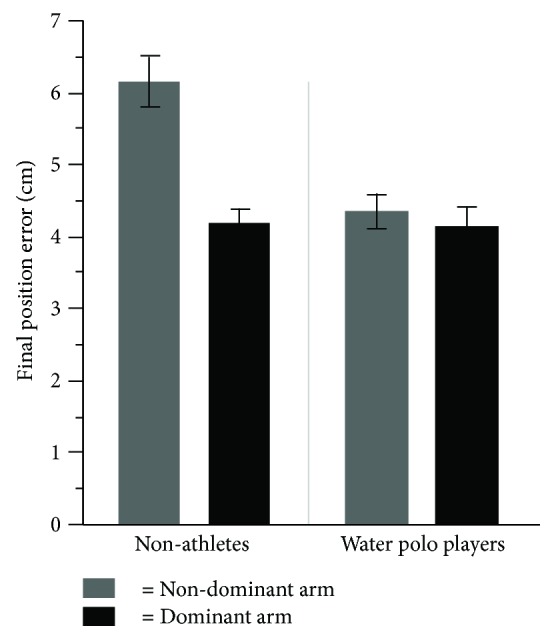
The average magnitude of the final position error (FPE) for the dominant and the nondominant arms for water polo players and nonathletes.

The result of the statistical analysis for the FPE displayed a significant difference between the group and arms (*F*(1, 24) = 9.61, *p* = .004, *η*^2^ = .48); arm and target direction interactions (*F*(1, 24) = 5.37, *p* = .02, *η*^2^ = .18); group main effect (*F*(1, 24) = 4.45, *p* = .04, *η*^2^ = .14); arm main effect (*F*(1, 24) = 14.78, *p* = .0008, *η*^2^ = .78); and target main effect (*F*(1, 24) = 22.03, *p* = .0001, *η*^2^ = .82). Post hoc analysis for group × arm interaction displayed (Figure 6) that both the nondominant and dominant arms of water polo players and the dominant arms of nonathletes had significantly better accuracy in comparison to the nondominant arms of nonathletes (*p* < .05). Post hoc analysis for arm × target directions showed that the dominant arms performed significantly better accuracy in target directions of 60 and 90 degrees in comparison to the nondominant arms (*p* < .05). The group main effect showed that water polo players overall had better accuracy in comparison to nonathletes (*p* < .05). The arm main effect also displayed that the dominant arms overall had significantly less FPE than the nondominant arms did (*p* < .05). The target main effect showed that reaches to the 30-degree target had better accuracy in comparison to 60- and 90-degree targets.

### 3.3. Hand Path Deviation from Linearity (HPDL)

**Figure 7:**
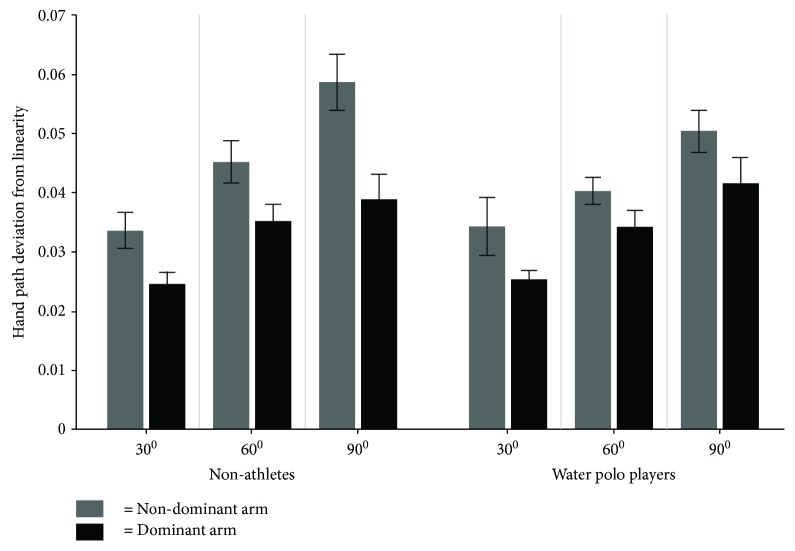
The average magnitude of the hand path deviation from linearity (HPDL) for each target direction for water polo players and nonathletes.

The average magnitude of the hand path deviation from linearity (HPDL) for each target direction for water polo players and nonathletes is displayed in Figure 7. As can be seen, the nondominant arm of both water polo players and nonathletes showed more HPDL in comparison to the dominant arm for all three target directions.

The result of the three-way mixed model ANOVA displayed only a significant main effect for the arm (*F*(1, 24) = 31.59, *p* = .0001, *η*^2^ = .76) and target (*F*(1, 24) = 154.02, *p* = .0001, *η*^2^ = .82). The movements with the dominant arm had significantly less curvature than those with the nondominant arm (*p* < .05). The main effect of the target revealed that the target located at 30 degrees was reached with movements of less curvature than targets located at 60 and 90 degrees. As the reaches to the target located at 30 degrees were mostly done with a single-joint movement, they could be done with movements of less curvature in comparison to the other targets.

### 3.4. Reaction Time

**Figure 8:**
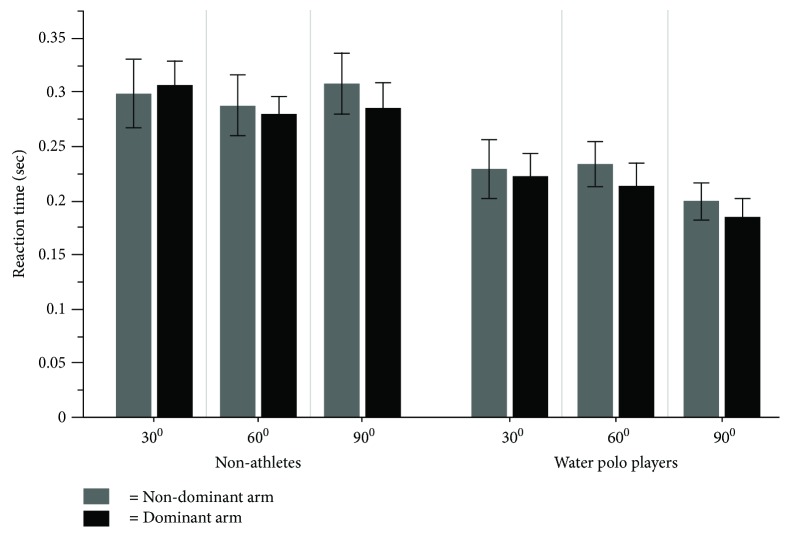
The average magnitude of the reaction time (RT) for each target direction for water polo players and nonathletes between the dominant and nondominant arms.

The average magnitude of the reaction times (RTs) for each target direction for water polo players and nonathletes between the dominant and nondominant arm is displayed in Figure 8. As can be seen, both arms had very similar RT for all targets for both groups. However, RT for water polo players is faster than that of nonathletes for all target directions.

**Figure 9:**
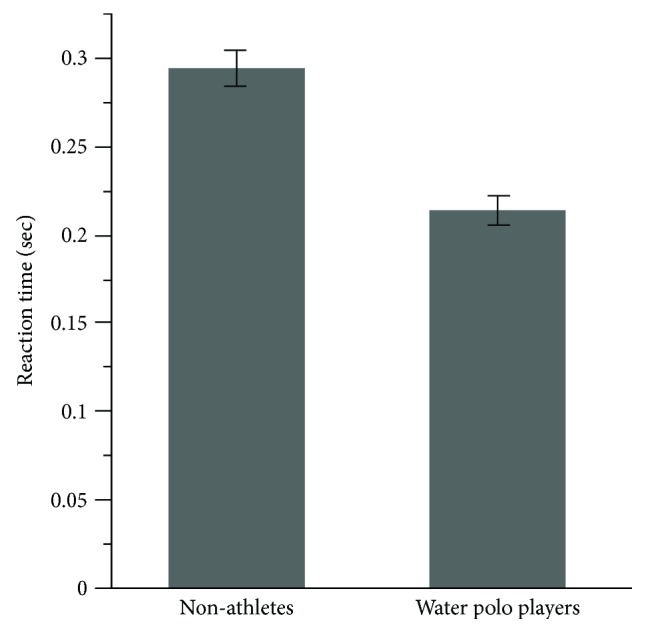
The average magnitude of the reaction time (RT) for water polo players and nonathletes.

The result of the three-way mixed model ANOVA for the RT displayed only a significant main effect for the group (*F*(1, 24) = 9.63, *p* = .004, *η*^2^ = .49) and target (*F*(1, 24) = 4.48, *p* = .04, *η*^2^ = .16). Water polo players (*M* = 0.213; SD = 0.07 m/s) had significantly faster RT than nonathletes (*M* = 0.293; SD = 0.09 m/s) (Figure 9, *p* < .05). The main effect of the targets revealed that the target located at 90 degrees was reached with faster RT than those located at 30 degrees.

## 4. Discussion and Conclusions

Previous studies reported that not only unimanual practice with the nondominant arm [8, 9] but also bimanual practice [10, 12, 14, 21] increased the performance of the dominant and nondominant arms for certain tasks. In a recent study, it was also stated that professional unimanual training dominantly with the right arm can improve the performance of both the right and left arms [13].

It has generally been accepted that athletes have better performance in some motor tasks, such as balance [22] and strength [23], than nonathletes do. Moreover, superior performance of athletes as a result of long-term practice has also been observed in some perceptual motor skills, such as reaction time [17]. In addition to scoring better in perceptual motor skills, athletes also displayed better motor performance in comparison to nonathletes [13, 14].

Similar to the findings reported in previous studies [6, 7], the nonathlete group in this study showed significant interlimb asymmetries in movement accuracy (FPE) and in movement quality (HPDL) such that right arm reaches were straighter and more accurate to targets across the workspace. The scores of the nonathletes were, generally, very similar to those in recent studies [6, 7, 13, 14]. However, the water polo player group in this study demonstrated more symmetric patterns of arm performance that were associated with substantially better accuracy. This may be an expected result, since the trainings with water polo players over the years required daily work on improving precision, related to the way of passing, shooting, and precise swimming.

Regarding the quality of movement of the nondominant arm of both groups, a larger hand path deviation from linearity (HPDL) was shown in relation to the dominant arm for all three directions of targets. Water polo is a sport where most of the manoeuvring with the ball is done with the dominant hand. The dominant hand is more burdened in the training process, so the result that the quality of the movement with the dominant hand is much greater than the nondominant one is somewhat expected.

Reaction time (RT) for both arms of water polo players was significantly faster than that for both arms of nonathletes, which is most likely the result of long-term training. Athletes have to simultaneously perform very intense short exercises and make decisions under strong time pressure [24]. Many researchers demonstrated that experienced players react more quickly than their less experienced counterparts and that there is significantly decreased RT in athletes as compared to nonathletes [25]. This phenomenon is evident in several sports, for example, basketball [26], table tennis [27], volleyball [28], badminton [29], and football [30]. Kramer et al. [31] found that participants who completed a six-month aerobic exercise program showed improvements in RT.

In the current study, we have also found faster RTs for water polo players for both their arms in comparison to nonathletes. In water polo, athletes must have quick arm reactions as they often block the opponent's shots on goal with both dominant and nondominant arms and those movements need to be explosive. Furthermore, reactions under water must be fast with both arms, which can explain the better RT of both arms in comparison to that of nonathletes in this study. As soon as a player in an attack situation notices any free space in the opponent's defence, he tries to shoot the ball to the goal; RT must be as fast as possible so that the action is efficient. Likewise, in that split second, the defence player attempts to raise his hand and blocks the shot; therefore, RT is important here as well. Such situations are constantly shifting with all players, which could explain their better RT than that of nonathletes. It has also been found that RT for the target located at 90 degrees is significantly faster in comparison to the targets located at 30 degrees. This is logical because water polo players mainly shoot towards the goal with an arm that is over the head and at approximately 90 degrees to the surface of the water. Such situations are more common than other arm positions from which shots are made. The same situation is observed during defence attempts and during blocking attempts, provided that in such situations dominant and nondominant hands have been used alternately, depending on which side the opponent player with the ball is located. However, it is important for the trainers to train the players to block the shoots in different angles like 30 and 60 degrees. Therefore, this will provide the possibility of a more effective defence against some shots from the sides.

In water polo, the optimal technique is essential for improving performance. Akpinar [14] found that asymmetry of hand performance with rowers is reduced because both arms should coordinately work together to perform the technique efficiently. Thus, performing skillful strokes consistently requires expertise and is a precondition for the effective bimanual control of the arms. In this respect, the decreased interlimb difference found in the current study in water polo players may be a requirement for skillful performance for swimming, manoeuvring under water, and maintaining balance in the water as well as blocking kicks with the dominant and nondominant arms. Symmetrical performance of both body sides has also been found in taekwondo [32]. Taekwondo athletes displayed similar performance of the left and the right sides of their body in some tests of motor abilities (flexibility, strength, and explosive leg strength) and performance quality tests of two basic taekwondo techniques. This may imply that athletes need to improve both body sides to acquire proficiency in their sports.

Neurophysiological characteristics can define a superior performance of any athlete. When performing skilled movements under different conditions and in changing environments, the athlete's brain needs to adapt to various types of behaviour [33]. This can include perception, decision-making, motor preparation, and execution of movements. For instance, in order to perform a skilled movement, the nervous system needs to activate required motor unit(s) in a proper manner at the right time and in the correct sequence [34]. This statement suggests that neural activities in the athletes' brains are modified with the participation of long-term training activities [35]. Confirmation has been obtained from some studies showing changes and shifts in brain activation among both musicians [36] and athletes [37] due to long-term practice. Specifically, motor-related cortical potentials, reflecting the movement preparation process, were found to be shorter and smaller in athletes compared to nonathletes [38]. Thus, the brain can easily be shaped by long-term skill acquisition, which also can improve performance when carrying out another skill or movement, such as the one observed in the current study. The water polo players with improved neural activations can have a better perception, decision-making, motor preparation, and execution of movements for both arms in comparison to nonathletes. In the future, it will be interesting to study similar characteristics with athletes of a similar skill level across sports that do not require unimanual activity.

Motor performance symmetries found in this study with water polo players were similar to those of the previous studies by Akpinar et al. [13] and Akpinar [14]. Even though it has been previously stated in some studies that the effect of a short-term unimanual practice on motor performance of both arms can be different [39], it seems that long-term sport participation can improve both arms' performance in the case of reaching movements. In general, superior motor performance during simple motor tasks for athletes and musicians over nonelite performers has previously been reported [24, 25, 40]. In water polo, arms are used for different purposes: i.e., the left arm is used to help in swimming and maintaining balance and in defence, and the right arm is practiced extensively to wield the ball. This can thus explain the better perceptual and motor performance of water polo players with both their arms in comparison to the performance of nonathletes. It can also give guidance to their trainers what they have done well and also what they need to improve in the player's training process. Regarding the talent identification for water polo, trainers should consider these motor and perceptual parameters to select the beginners for this sport. This study showed the very intriguing result that elite water polo players had significantly better accuracy with significantly shorter RTs than nonathletes. It could be expected that a shorter RT of a reaching movement will likely make the movement less accurate; however, this was not the case in this study with the elite water polo players. Over the long-term training, players might improve the ability to perform fast reacted and accurate movements. Whether water polo players are less asymmetric because of practice in water polo or whether symmetry in performance is associated with an implicit advantage that might influence competitive selection for water polo cannot be conclusively addressed with the current study design.

The current study has a limitation for the selection of a control group; thus, future studies should focus on comparing perceptual and motor performance asymmetries in different types of sports to be able to understand the fundamental characteristics of sports. Longitudinal studies should also be conducted to investigate whether the decreased interlimb difference in water polo is a result of a long-term practice or whether it had existed before starting the sport. Moreover, in addition to the perceptual and motor performances, investigating athletes' neurophysiological background would be sufficient to make a connection between motor and neural mechanisms.

## Data Availability

The data used to support the findings of this study are available from the corresponding author upon request.

## Additional Points

*Consent for Publication*. The authors gave their consent for publication.
